# Experiences of nurse practitioners and medical practitioners working in collaborative practice models in primary healthcare in Australia – a multiple case study using mixed methods

**DOI:** 10.1186/s12875-016-0503-2

**Published:** 2016-07-29

**Authors:** Verena Schadewaldt, Elizabeth McInnes, Janet E. Hiller, Anne Gardner

**Affiliations:** 1 Faculty of Health Sciences, School of Nursing Midwifery and Paramedicine, Australian Catholic University, Melbourne, Australia; 2 Nursing Research Institute, St Vincent’s Health Australia/Australian Catholic University, Sydney, Australia; 3 School of Health Sciences, Faculty of Health, Arts and Design, Swinburne University of Technology, Melbourne, Australia; 4 School of Public Health, University of Adelaide, Adelaide, Australia; 5 Faculty of Health Sciences, School of Nursing, Midwifery and Paramedicine, Australian Catholic University, Canberra, Australia; 6 James Cook University, Townsville, Australia

**Keywords:** Nurse practitioners, Primary health care, Physician-nurse-relation, Health policy, Collaboration

## Abstract

**Background:**

In 2010 policy changes were introduced to the Australian healthcare system that granted nurse practitioners access to the public health insurance scheme (Medicare) subject to a collaborative arrangement with a medical practitioner. These changes facilitated nurse practitioner practice in primary healthcare settings. This study investigated the experiences and perceptions of nurse practitioners and medical practitioners who worked together under the new policies and aimed to identify enablers of collaborative practice models.

**Methods:**

A multiple case study of five primary healthcare sites was undertaken, applying mixed methods research. Six nurse practitioners, 13 medical practitioners and three practice managers participated in the study. Data were collected through direct observations, documents and semi-structured interviews as well as questionnaires including validated scales to measure the level of collaboration, satisfaction with collaboration and beliefs in the benefits of collaboration. Thematic analysis was undertaken for qualitative data from interviews, observations and documents, followed by deductive analysis whereby thematic categories were compared to two theoretical models of collaboration. Questionnaire responses were summarised using descriptive statistics.

**Results:**

Using the scale measurements, nurse practitioners and medical practitioners reported high levels of collaboration, were highly satisfied with their collaborative relationship and strongly believed that collaboration benefited the patient. The three themes developed from qualitative data showed a more complex and nuanced picture: 1) Structures such as government policy requirements and local infrastructure disadvantaged nurse practitioners financially and professionally in collaborative practice models; 2) Participants experienced the influence and consequences of individual role enactment through the co-existence of overlapping, complementary, traditional and emerging roles, which blurred perceptions of legal liability and reimbursement for shared patient care; 3) Nurse practitioners’ and medical practitioners’ adjustment to new routines and facilitating the collaborative work relied on the willingness and personal commitment of individuals.

**Conclusions:**

Findings of this study suggest that the willingness of practitioners and their individual relationships partially overcame the effect of system restrictions. However, strategic support from healthcare reform decision-makers is needed to strengthen nurse practitioner positions and ensure the sustainability of collaborative practice models in primary healthcare.

**Electronic supplementary material:**

The online version of this article (doi:10.1186/s12875-016-0503-2) contains supplementary material, which is available to authorized users.

## Background

Over the last 15 years, nurse practitioners (NPs) have become part of the Australian primary health care (PHC) sector. While the USA and Canada have utilised NPs in the healthcare system since 1965 the first NPs in Australia were formally authorised to practice in 2000 [1]. By March 2015 there were 1214 endorsed NPs, reflecting modest diffusion through the health care system [2]. Australian NPs are registered nurses with a minimum educational level of a Master’s degree [3] and endorsement is regulated through the Australian Health Professional Regulation Agency (AHPRA). This endorsement includes the ability to prescribe. While endorsement is regulated through a national body, state-level legislation *regulates* prescribing rights [4].

A systematic review of US-based studies identified that NPs in PHC settings achieve excellent outcomes for their patients in regard to risk factor management, patient satisfaction, functional health status and hospitalisation rates [5]. A broader literature review including Australian and international literature confirmed that nurses and NPs in PHC can effectively and safely provide healthcare to patients [6]. Consequently they can contribute to solutions for current healthcare service delivery issues, which have occurred from escalating demands with an ageing population, an overall population growth, a rise in chronic diseases, an increase of healthcare service costs and workforce shortages [7]. However, a recent World Health Organisation (WHO) report on the healthcare workforce highlighted the underutilisation of advanced health practitioners, such as NPs, in addressing current healthcare issues world wide [8]. A review of NP implementation processes internationally highlighted a number of reasons for the underutilisation of NPs, such as a lack of knowledge of the NPs’ scope of practice, non-recognition of their skills and lack of financial and organisational support for their implementation [9].

Primary healthcare in Australia offers the first point of contact for patients in the community and is based on a mixed funding model that includes funding from government programmes, direct payments from patients and private health funds [10]. Medicare, the government-funded public health insurance scheme subsidises a wide range of health services listed on the Medicare Benefits Schedule (MBS) and prescription medicines listed on the Pharmaceutical Benefits Scheme (PBS) [11]. Designated healthcare providers such as MPs, NPs, radiologists and allied health professionals can choose to charge the government-subsidised fee or charge an additional fee that the patient has to pay privately. Healthcare costs for PHC services in Australia account for 36.1 % of the total healthcare expenditure [11].

Since 2010, policy amendments to the National Health Act 1953 authorised NPs in Australia to prescribe medication as listed in the PBS and access the MBS [12, 13], which facilitated their implementation as PHC providers. Similar to some states in the USA [14], it is a prerequisite by Australian law for NPs to enter a collaborative arrangement with a MP in order to access Medicare subsidy schemes [15]. Table 1 presents four options of determining a collaborative arrangement and the frequency of their occurrence in practice.

**Table 1:** Collaborative Arrangements - forms and occurrence in practice

Forms of collaborative arrangements^a^	Percentage of collaborative arrangements (ACNP member survey) [93]
(1) a written agreement about collaborative practice between the NP and the MP exists, or	51.0 %
(2) the NP is employed or engaged by a MP or an institution that employs or engages MPs, or	37.8 %
(3) a patient is referred to the NP by a MP, or	8.1 %
(4) an agreement about collaborative care for an individual patient is stated in the patient’s clinical notes by the NP.	2.7 %

^a^National Health (Collaborative Arrangements for Nurse Practitioners) Determination [15], enabled by the *Health Insurance Regulations 1975*, section 2 F

*NP* Nurse Practitioner, *MP* Medical Practitioner, *ACNP* Australian College of Nurse Practitioners

National and international empirical evidence from interviews and surveys of NPs indicate that NP positions evolve where they receive support from MPs [16–18]. Support from MPs for the implementation of NPs is crucial with the requirement of collaborative arrangements [17, 19]. However, multiple factors can hinder or enable the establishment of collaborative practice models. An integrative review of collaboration between NPs and MPs in PHC identified numerous barriers to successful and satisfying collaborative work arrangements globally [20]. These barriers included interpersonal differences, system structures such as legislation and organisational protocols, a lack of clarity as to professional roles and financial aspects of collaboration [20]. The review identified no published Australian studies.

Collaboration is influenced and shaped by system structures, organisational arrangements and interpersonal relationships [21]. At the level of system structures, American economists identified the introduction of NPs to healthcare systems as a “disruptive innovation” [22, 23]. Disruptive innovations offer “cheaper, simpler, more convenient […] services” ([22], p. 2). Nurse practitioners fulfil these criteria because they are able to diagnose and treat patients and provide cheaper healthcare services without compromising on quality and thus appeal to customers with unmet healthcare needs [23]. As a consequence, NPs offer services that have traditionally been regarded as part of a medical practitioner’s work spectrum and “disrupt” existing service structures [22].

The addition of NPs to PHC creates an overlap of the scope of practice with MPs requiring the re-negotiation of professional boundaries and roles [24], which can affect interpersonal relationships. Historically the relationship between nurses and medical practitioners has been hierarchical [25, 26]. Conditions that foster power imbalances between nurses and MPs and a “structural embeddedness of medical dominance” ([27], p. 482) continue to exist in healthcare systems of North America and the UK [28]. In Australia, the slow implementation of NPs was in part ascribed to “behind-the-scenes influence” ([29], p. 428) of the medical profession.

The outcomes of international research confirm the complexity of collaboration and therefore the findings cannot be transferred from one setting to another without understanding these complexities. Anecdotally there is controversy around collaborative arrangements and NP access to funding schemes in Australia [30–32]. The consequences of policy amendments regarding collaborative arrangements between NPs and MPs as a prerequisite for NP access to Medicare subsidy schemes are under researched in Australia [33]. This study is the first to report on experiences of Australian NPs and MPs who work together in collaborative practice models.

The aim of this study was to identify the experiences and perceptions of NPs and MPs working collaboratively in PHC settings in Australia following amendments to existing policies. The specific research questions were: What are Australian NPs’ and MPs’ experiences and perceptions of collaborative practice in PHC under new legal policies? What factors enable collaborative practice models to function?

## Methods

This research comprised multiple case studies employing mixed methods research. A case study design was chosen because it is highly suitable for identifying the particularities and complexities of a phenomenon in everyday contexts [34, 35]. For an investigation of collaboration between NPs and MPs the contextual conditions in which collaboration occurred were considered very important to capture, as they might influence how collaborative practice models were realised. The inclusion of multiple cases in this study served to generate a more comprehensive understanding of the issue under investigation and provide a more powerful and robust basis for conclusions than a single case study [36, 37].

Within the multiple case study design mixed methods research (MMR) was applied [38] to triangulate methods and data sources for data enrichment, corroboration or identification of contradictions [39, 40]. This study was based on a qualitative core component including interviews, non-participant observations and documentary data that was supported with a quantitative component comprising a questionnaire [41].

Considering the available evidence from international research two models of collaboration provided a theoretical framework to inform some questions of the interview schedule and parts of the data analysis. These models were selected on the basis that the *Conceptual Model of Collaborative Nurse-Physician Interaction* was the only model to specifically focus on collaboration between nurses and MPs [42]; and the *Structuration Model of Collaboration* was based on extensive research and applied in multidisciplinary PHC settings [43]. The models present influencing factors of collaboration between health professionals including interpersonal, organisational and systemic dimensions. Table 2 presents the 17 combined dimensions of both models and shows where dimensions overlap and complement each other.

**Table 2:** Dimensions of the Structuration Model of Collaboration and the Model of Nurse-Physician Interaction

	Dimension	Model
1	Mutual trust and respect	C, S
2	Formalisation tools (policies, protocols, agreements)	C, S
3	Communication/behaviour tendencies/Information exchange	C, S
4	Compatible role perceptions/mutual acquaintanceship	C, S
5	Joint goal setting and decision making	C, S
6	Complementary management of influencing variables/Client-centred orientation vs other allegiances	C, S
7	Conditions of power symmetry	C
8	Traditions of professionalization	C
9	Traditional gender/role norms	C
10	Personal attitudes	C
11	Complexity of care environment (the higher, the more collaboration)	C
12	Prevalent social reality	C
13	Nursing/medical school curricula	C
14	Support for innovation	S
15	Connectivity (opportunities for discussion and adjustment of coordination problems, for example information and feedback systems, meetings, committees etc.	S
16	Centrality (authorities that provide clear directions that foster collaboration, inherits a strategic and political role)	S
17	Leadership (local person)	S

*C* Conceptual Model of Collaborative Nurse-Physician Interaction [42]

*S* Structuration Model of Collaboration [43]

### Recruitment and selection of sites

Recruitment of sites occurred from August 2012 to May 2013 through emailing a research invitation to members of nursing and medical organisations, calling potential PHC sites where NPs worked with MPs, and through publicising the study at NP workshops. Throughout the recruitment phase a snowball sampling technique was applied to identify further potential study sites [44] by asking NPs and MPs to promote the study to interested nursing and medical colleagues.

Potential sites were screened against selection criteria (Table 3). Once eligibility was confirmed, a telephone conference was undertaken with potential site staff to identify site characteristics such as practice size, practice type (public or private) location (urban or remote), PHC specialty and type of collaborative arrangement. Sites were purposefully selected considering maximum variation of these site characteristics. Data saturation was ensured by successively recruiting cases to the study. Following data collection at each site, preliminary analysis was initiated before the next site was visited for data collection. Once information and preliminary themes became repetitive, no further sites were recruited to the study. Prior to data collection written informed consent was sought from all study participants.

**Table 3:** Selection criteria

**Inclusion criteria**
• Primary healthcare setting
• NP and MP registered with AHPRA for at least 6 months
• NP endorsed as NP for at least 6 months
• NP and MP working together for at least 6 months for at least 1 day per week
• Both NP and MP needed to be willing to participate in the study
**Exclusion criteria**
• Secondary/tertiary healthcare setting
• Sites with practice nurses or NP candidates who were not endorsed as NPs yet
• Participants who have not worked together for a minimum of 6 months
• Sites with complicated travelling logistics that would have exceeded the study budget

*NP* Nurse Practitioner, *MP* Medical Practitioner, *AHPRA* Australian Health Practitioner Regulation Agency

### Data collection and analysis

Data collection was undertaken in three phases involving four data sources. Details about data collection methods and analysis have been reported previously [45] and are summarised here.

1. Non-participant observations of NPs and MPs were undertaken to capture collaborative behaviour and interactions; communication patterns and organisational and clinical context.
2. Nurse practitioners and MPs were asked to complete a questionnaire to collect demographic information. The questionnaire included three validated scales. Two scales were based on a provider collaboration survey, developed for NP-MP collaboration in Canadian primary healthcare settings to measure the experience with collaboration (9 items, 6-point Likert scale) and satisfaction with the collaborative relationship (15 items, 6-point Likert scale) [46]. In this study we used the expanded and modified versions by Donald et al. [47]. Both scales were pilot-tested for content validity, relevance and understandability by the original authors [46] in Canadian PHC settings. The modified versions by Donald et al. F Donald [47] were also tested for construct validity by comparing each of the scales with a single general question. This resulted in Spearman’s *r* = 0.89, *p* < 0.001 for the scale measuring experience with current collaboration and *r* = 0.91, *p* < 0.001 for the scale on satisfaction with collaboration [47], indicating very good construct validity.
  The third scale measured beliefs in the benefits of collaboration (5 items, 5-point Likert scale) developed as part of a survey to identify interprofessional processes in teams [48]. The scale had high reliability (Cronbach’s α coefficient of 0.91). Factor analysis showed sufficient loading of the items on a single factor confirming high construct validity [48].
  Higher scores indicated stronger perceptions of collaboration on all three scales. Permission to use the scales was granted from the original authors. The questionnaire was pilot-tested by a group of health academics, NPs and MPs.
3. Individual face-to-face interviews were conducted with NPs and MPs using a semi-structured interview guide (see Additional files 1, 2 and 3) to identify personal experiences of barriers and facilitators to collaborative working, perceptions on shared decision-making, autonomy and supervision as well as views on the legal requirement of collaborative arrangements. Where these positions existed, practice managers (PMs) were asked to participate in an interview to capture their perspective on the collaboration between NP and MP.
4. Throughout the data collection period at each site, practice documents stating the collaborative arrangements, the scope of practice of the NP and flyers for patients explaining the NP role within the practice were collected to gain further insights in work mechanisms and roles that were defined in writing in these documents.

Data analysis occurred in several stages. Index scores of scales and demographic data were analysed using descriptive statistics. After consultation with a statistician statistical analysis was limited to descriptive statistics, which is a minor revision to the protocol [45]. Analysis of qualitative data was informed by the thematic analysis approach suggested by Braun and Clarke [49]. QSR International NVivo 10 software was used to assist data management and analysis. Braun and Clarke distinguish ‘data-driven’ (inductive) or ‘theory-driven’ (deductive) coding, which was preferable for this study to generate codes based on participant meaning first and then allow for comparison with the current theoretical models [49].

The inductive approach of qualitative data analysis identified new codes inherent to the participants and sites of this study. To allow comparison of the participants’ views (interviews), the researcher’s observations and documents describing the collaborative practice (practice documents), the three data types were coded separately and collapsed into thematic categories. Thematic categories from interviews and observations were compared through triangulation and summarised in themes. Reasons for differences and commonalities of codes are reported in the narrative of the developed themes. We drew on codes from the document analysis when they were useful to clarify or support themes. In a second step of triangulation, questionnaire results were woven together with themes at the point of data interpretation and are highlighted where they supported or contradicted qualitative findings [50].

A theory-driven and deductive approach of data analysis then assisted with determining how close the data set of this study was to existing international models by comparing the 17 combined dimensions of influence of collaboration [42, 43] (Table 2) with the empirically derived codes and categories in NVivo.

Ethics approval for this study was granted by the Human Research Ethics Committee of the Australian Catholic University (No. 2012 207 V). Stringent quality measures were applied to establish credibility and trustworthiness of findings [51]. These included the adherence to a research protocol [45], the use of a research diary, data triangulation, and comparison with existing theoretical frameworks. Potential influences of researchers on the study process were discussed to minimise bias. All authors are health services researchers, three with a nursing background.

## Results

Of 13 eligible sites, five were selected including 22 participants comprising six NPs, 13 MPs and three PMs considering variation of site characteristics and availability within the study period (Table 4). Site locations included country towns with a population under 2000, larger towns with 200,000-300,000 residents and cities with populations ranging from 1 to 4 million. Four sites were privately owned practices while the community centre was publicly funded. In total, data collection included 143 h of non-participant observation (varying from 3 to 10 days per site), a return of 18 questionnaires (95 % return rate), compilation of 12 practice documents and 21 interviews ranging from 16 to 60 min in duration.

**Table 4:** Study Sample Characteristics

Sites	
Practices	4 private practices, 1 community centre
Locations	New South Wales, South Australia, Tasmania, Victoria
NPs per practice	1–2
MPs per practice	2–20
Individual participants	
Nurse Practitioners	6, all female
NP specialties	PHC, cardiology, aged care, drug and alcohol withdrawal
Working as NP (median, range)	2.0 years (0.5–11.5)
Medical Practitioners	13, four female
MP specialties	General practice/PHC, cardiology, gerontopsychology
Experience in PHC (median, range)	NPs: 8.75 years (1.2–15)
	MPs: 13.0 years (2.3–34)
Practice Managers	3, all female

*NP* Nurse Practitioner, *MP* Medical Practitioner, *PHC* Primary Healthcare

The organisational context and working structures differed at each site. Practice size ranged from large practices at several locations and more than 20 MPs to small practices with 2 MPs at one location. At some sites NPs worked most of the time in the community whereas other NPs worked in consulting rooms at the practice. Practice managers managed the four private practices. At the community centre the NP ran the centre in her position as nurse unit manager and MPs were not consistently present on site but visited on a daily basis. Not all MPs in larger practices worked with the NP and not all MPs were participants in this study.

In general, separate healthcare consultations of NPs and MPs prevailed at all sites with NPs and MPs operating as autonomous health professionals. The collaborative character of the practice models only emerged when mutual patients were discussed or referred to another health professional. Information exchange about patient care occurred through meetings, internal messaging systems, phone calls and referral letters. Face-to-face contact between NPs and MPs at sites ranged from daily to weekly encounters.

### Questionnaire results

High scores on all scales indicated positive perceptions in the descriptive analysis. Median index scores of the three scales showed 1) NP and MP groups strongly believed that collaboration was beneficial for patients; 2) they experienced high levels of collaboration and 3) were highly satisfied with their collaborative relationship (Table 5).

**Table 5:** Index Scores of three Scales (Median and Range)

Index scores	Median^a^ [Range]	
	NPs	MPs
Beliefs in the benefits of collaboration	5.0 [4.2–5.0]	4.7 [3.3–5.0]
Experience with current collaboration	4.9 [4.7–5.3]	5.4 [2.7–6.0]
Satisfaction with current collaboration	5.1 [4.2–5.5]	5.4 [2.6–6.0]

^a^Median of means of individual responses, *NP* Nurse Practitioner, *MP* Medical Practitioner

The data revealed a greater variation among MP responses reflected in a wider range for all three scales. Instead of interquartile ranges, the minimum and maximum are presented for all scales to reflect the full range of responses in this small sample.

Results from thematic analysis of interview and non-participant observation data are presented in three main themes.

### Influence of system structures

This theme reports challenges of working in collaborative practice models due to healthcare system structures, policies and also infrastructure at practice level. One of the major constraints to establish and maintain collaborative practice models was the way Medicare reimbursed NPs. While NPs, MPs and PMs valued NPs’ access to Medicare, they critiqued the current reimbursement rates and available MBS items for NPs as insufficient and unfair. Nurse practitioners in private practice can use four professional attendance MBS items for patient consultations and a limited number of diagnostic test items [13]. For example, electrocardiography is a common investigation for NPs caring for cardiac patients, but it would incur the patient a private fee if ordered by the NP rather than the MP. In these cases, care needed to be escalated to the MP for ordering the investigations once the NP completed the initial patient assessment. “*Why do I see it not as equal? Because… […] they [MPs] have the capacity to request more investigations than we do. I think, our practice [services that are covered by MBS items] is somewhat restricted by what Medicare says” (NP).* Another example refers to ‘Chronic disease management plans’ for a joint approach to patient care that required MPs to sign off on care plans, resulting in reimbursement going to MPs. However, typically the NP spent most of the time with the patient for assessment and planning.

In general, NPs and MPs commented that the fee-for-service (FFS) structure of Medicare lacked adequate financial compensation for health professionals discussing mutual patients. These discussions were common occurrences and considered important for a complementary approach to a person’s care. *“If there needs to be feedback to [NP name] or [NP name] needs to talk to me we have to do that in our own time. And that can be a significant amount of time during the day you don’t get paid for” (MP).*

In addition to Medicare policies, the legal determination of collaborative arrangements impacted on collaborative practice between NPs and MPs. In our study, four of five practice settings had a written agreement [52]. In the community centre, no written arrangement existed but the legal determination was fulfilled because the organisation for which the NP worked sub-contracted MPs.

Some NPs and MPs perceived collaborative arrangements as positive because they considered it a safety net, which supported NP practice when a patient scenario required a second opinion or transfer of care through the availability of a MP*. “I do find it helpful. I think it’s safe. I think that’s the biggest issue, the fact that you know you’ve always got that backup” (NP).* On the other hand, NPs critiqued the legal formalisation of collaboration. They considered it common sense to consult with another health professional when they needed a second opinion. Collaborative arrangements are *“a sore point that nurse practitioners fought not to have formal [legally required], because we feel we would refer anyway if we find something outside our scope” (NP).* One NP reported that she was unable to establish a NP-led clinic because MPs declined to engage in a collaborative arrangement.

These policies and regulations weakened the NPs’ position as legitimate healthcare providers within the collaborative practice. Difficulties in generating income decreased their chances of finding a practice that was willing to employ them. *“In a private GP practice, at this stage, [we] couldn’t make enough money to fund ourselves or make it worthwhile for them [MPs] to fund us” (NP).* The NP’s limited ability to contribute to practice income reinforced uncertainty about the financial sustainability of NPs, which may impede the establishment of collaborative practice models because potential loss of income prompted MPs’ concerns. Nurse practitioners reported that they were not entitled to demand their own office because they could not contribute sufficient income to the practice. Consequently, existing healthcare system regulations created a hierarchical, as opposed to balanced, professional relationship and contradicted the definition of ideal collaboration that emphasises equality, shared power and interdependency [53, 54].

At a practice level, a major challenge mentioned by NPs and MPs was a lack of dedicated time to actually collaborate, that is, discuss shared patient cases, which was also identified in the questionnaire. Most participants would have liked time for more face-to-face meetings, but the busyness of the practice did not allow for this.

*“We don’t have a system here where there is protected time for us to sit down with the practitioner and be able to communicate the concerns and that sort of thing. It sort of ends up being something in the hallway: ‘Oh by the way, I saw that person and this and that’” (MP).*

Conversations were more sporadic at three sites where the NP and MP were not on site together on a regular basis suggesting that physical proximity increased the chances of communication and collaboration. At other sites, the lack of a communal room and facilities impeded opportunities for communication, a defining principle of collaboration. *NP has lunch, standing. There are no chairs to sit. Some admin staff are in the kitchen. There is not much time for conversation. Everyone is standing while eating (Observation).*

Interview data highlighted differing perceptions about the importance of face-to-face meetings. At one site a NP was scheduling her time in between home visits according to the availability of the MPs at the practice. She said: *“I’ll catch them informally again, I hover (laughs), make myself available, when I know they have a break” (NP).* One MP also valued this time of direct exchange but noted: *“It just seems to happen that we meet there” (MP).* The MP seemed unaware of the significance of this meeting to the NP, not realising that the NP had actively tried to be around to meet her. For the MP the meetings seemed a convenience, for the NP a priority when working together.

Integrating NPs into existing infrastructure posed a challenge. Due to a shortage of rooms some NPs and MPs frequently changed offices and some NPs used MP consulting rooms. Nurse practitioners stored materials and utensils in a box or movable storage trolley to adjust to this situation. One NP had no consulting room allocated within the practice because she worked mainly in nursing homes or visited patients at home. The lack of designated workspace caused uncertainty about her availability amongst the collaborating MPs because she only returned to the practice sporadically and used different locations within the practice to complete administrative work. I observed her working with a laptop on her knees, surrounded by other staff.

*9.30 am – Communal area: In a corner is a 1 m*^*2*^*small desk with computer and printer. The NP wanted to print something there, but it is occupied by someone […] Standing, she is going through her papers, makes phone calls, operating in the middle of the room. There is no privacy (Observation).*

In addition to physical integration, interview statements and observations revealed that NPs experienced pressure to find and assert their position within the existing system. Some MPs were sceptical as to whether NP care differed from care provided by MPs. “*[Is it] just another way […] of doing something that GPs are already doing?” (MP).* Difficulties with integrating a new health professional were also reflected in the NPs’ negative experiences with dismissive MPs, including those not participating in this study or external to the practice setting. Consequently, NPs wanted to prove their worth, for example, one NP reported a patient satisfaction survey she initiated and in which she received very good feedback. That was important for her because *“that was something I could demonstrate to the practice manager and the board that what I am doing is worthwhile” (NP).* This pressure to physically and professionally integrate was not observed for MPs given their long-standing history as PHC professionals.

### Influence and consequences of individual role enactment

The second theme reflects on the team roles of NPs and MPs and how NPs and MPs operationalised their work arrangements with complementary roles. For clarity of reading, this theme is divided into three sub-themes.

#### Influence of NP autonomy

Role enactment refers to the process of participants familiarising themselves with their roles as collaborating colleagues and performing their specific roles within the team. The NPs’ level of autonomy led to an expansion of their scope of practice and in some cases caused an overlap with the scope of practice of MPs, which led to blurred professional roles. *“I know that she does some of the work that I would otherwise be doing” (MP)*. The lack of differentiation of the NP role from the MP role in practice occurred despite clear statements about the NP’s role in practice documents. Understanding the new role of NPs was complicated because NPs had previously been in practice nurse roles with the same MPs and still retained some practice nurse functions. In Australia, practice nurses are enrolled or registered nurses who can autonomously see patients but commonly under the supervision of a general practitioner [55]. In comparison to the NP, a practice nurse participates in many procedures in an assisting capacity and cannot access the Medicare subsidy schemes.

The difficulty to clearly define the NP role may have contributed to some MPs’ ambivalence about NP autonomy. Some MPs expressed a general concern about fragmentation of care and appropriate decision-making by NPs. *“I always worry, if there was something missed” (MP).* On the other side, some MPs strongly supported an autonomous NP role and some MPs expected NPs to take more responsibility by making autonomous decisions about patient care. *“I would expect [NP name] to make the actual [patient] management decisions” (MP).*

Nurse practitioners also valued their autonomy but applying it in practice was shaped by two factors; their level of confidence to make autonomous decisions and policy restrictions that required the MPs’ involvement as outlined in the first theme. A MP commented on the questionnaire: *Some NPs can’t or don’t want to make a full decision on her/his scope (MP).*

The ways that NPs exercised and MPs accepted NP autonomy influenced referral and consultation patterns between NPs and MPs. Researcher observations showed that MPs mostly *referred* patients to the NP, that is they passed on the patient for an additional consultation with the NP; while NPs in addition to referrals *consulted* MPs, that is they sought advice from MPs while the patient was with them. While patient referrals to the NP were perceived as an alleviation of workload for MPs, one-sided consultation patterns of NPs caused interruptions to the work of both practitioners. We observed waiting times between 1 and 25 min until a MP was free to assist the NP who was waiting with the patient in her office. Medical practitioners also had to interrupt their workflow and sometimes added an additional patient from the NP to their already full schedule. *“I was really busy and then sometimes, you know, extra referrals from the nurse practitioner can be a little bit too much because it is an extra appointment” (MP).*

#### Perceptions on reimbursement and legal liability

The joint involvement of practitioners for some patients highlighted that autonomous and collaborative roles of NPs and MPs co-existed. The co-existence of roles affected perceptions of who should be reimbursed and who was legally liable for shared patient care. In regard to reimbursement, NPs consulting the MP for less than a minute to ask a question was a common occurrence but one MP emphasised: *“We don’t have a way to bill that” (MP).* Some NPs were concerned that MPs were not reimbursed for these times. Other NPs considered it inappropriate for the MP to bill the patient for a short consultation, which was possible when the MP had joined the NP’s session with the patient, because these NPs believed discussing patient issues was a courtesy among colleagues.

*“The billing thing is, I think, is the biggest issue. I am troubled with that sometimes and the fact that I don’t think somebody walking in the room for two seconds saying ‘hello’ warrants an item number. And I think some doctors here would dispute that, because they have seen the patient. […] But I don’t think that’s fair on Medicare or the patient” (NP).*

Despite Medicare policies on what constitutes a consultation [56] there was room for interpretation, depending on whether the MP considered herself as the reimbursable practitioner or an advice-giving colleague. For both NPs and MPs, reimbursement claims relied on an interpretation of their role; that is which of the practitioners considered themselves reimbursable for a joint patient consultation.

Professional guidelines issued by medical and nursing boards in Australia clearly state that each health professional is responsible for his or her own actions and decisions [3, 57]. Practice experience showed that medico-legal liability was less clear when patient care was shared between NP and MP. Contrasting perceptions on liability were identified in interviews.

The majority of MPs but none of the NPs considered MPs as *“ultimately responsible” (MP),* even for those patients cared for by the NP alone. Some MPs thought that the collaborative arrangements served to establish legal liability within the collaborative practice, assigning ultimate responsibility to MPs. One MP stated that collaborative arrangements *“made us, the GPs, much happier about our risk”,* reflecting the assumption of some MPs that the legal determination addressed professional liability. However, the determination does not stipulate the assignment of liability, which is supported by the fact that it can be a verbal agreement.

Nurse practitioners and some MPs considered responsibility lay with the practitioner primarily caring for a patient. *“If I write the order then I would be responsible totally for my actions and if the GP writes the order then they would be totally responsible” (NP).* However, system requirements for NPs to obtain a signature from the MP for certain procedures destabilised the concept of being accountable for one’s own practice. For example, it was the NP’s decision to refer a patient to mental health services, but the MP became the person responsible because she had to sign the referral form. Without Medicare restrictions the NP could have placed the order and lines of liability would not have been blurred.

Some practitioners agreed that they shared legal liability. Shared responsibility came into effect when a practitioner gave advice to another practitioner and this was recorded in the patient notes and incorporated in the patient’s care. However, for MPs it was difficult to know if the “quick” advice in the corridor would be used and regarded as MP involvement in patient care and consequently if it made them legally liable for this patient. Therefore, MPs preferred to be either fully involved in patient care and see the patient or not be included at all. *“If she doesn’t refer [to] me I don’t want to know anything about her patient […]. If she refers a patient to me, then I want to know everything. I want to take over” (MP).*

#### Working in complementary roles

The blurring of roles and responsibilities was not observed to negatively affect direct patient care because the NP and MP worked either in separate autonomous patient consultations or worked with complementary skills for shared patient consultations. For most patient consultations, interview and observation data clearly showed NPs and MPs providing complete episodes of care without collaborative interaction. *“It’s a separate process. I usually make my decisions and if she sees a patient she makes her decisions” (MP).* For these autonomous consultations NPs applied what has traditionally been seen as nursing and medical skills whereas for shared episodes of care NPs tended to focus on nursing care and MPs on medical care so that roles complemented each other. Medical practitioners perceived that working in this complementary manner enhanced collaborative practice: *“It just adds another dimension to your understanding of the patient” (MP).* In particular the educational role of NPs, who must also be registered nurses in Australia, complemented MP consultations that focused on diagnostics and medication.

*“So I think, that [diagnosing] is the cardiologists’ role and from then on they can come to me for all the management issues, you know, education, the lifestyle, the action plans, all the other issues that revolve around chronic illness” (NP).*

The complementarity of roles was also evident when NPs and MPs returned to traditional role patterns, with MPs as the dominant care provider and NPs functioning in a subordinate role as practice nurses. Self-perpetuating traditions of MPs “owning” patients and making final decisions were evident in statements of participants: *“But there still is a hierarchy where… In general practice, I feel like the patients still belong to one of the doctors” (NP)*. This attitude was also expressed by a practice manager who explained that the MPs could decide if they wanted to squeeze in an acute patient or if the patient should be booked with the NP instead. It implied that MPs had the primary choice of patients.

Language used by MPs also revealed the existence of historical ways of thinking. Some MPs considered themselves as *“supervisor”*, describing the NPs as their *“right hand”* or talking about the NPs, who were all female in this sample, as *“girls”*. Often these statements were explicit acknowledgements of the NPs’ importance to patients and the additional value to the practice, particularly evident in the following statement. *“But these girls are helping out enormously in terms of patient load” (MP).* Therefore, this behaviour could be interpreted as a form of subconscious paternalism. The presence of traditional role patterns in day-to-day practice appeared to be accepted by NPs and MPs. This suggests that going back and forth between old and new roles, was part of the process of finding matching roles within the collaborative practice models.

### Making it work: adjustment to new routines

Practitioners developed strategies and abilities to successfully work together. Planning and preparation were required to arrange practicalities. At a practice level, these included developing a concept for the collaborative practice model and holding initial meetings to inform staff, clarify questions and dispel concerns. Preparations also needed to address space and equipment. *“So we had to put in a sink, change the curtain; change it into a clinical room. So it wasn’t just a matter of slotting someone in. We had to kind of make it happen” (PM).* Practice managers were identified as a resource for adjustments of practice infrastructure. They were involved in the organisation of team meetings, acted as moderator in case of conflicting interests and facilitated information flow between NPs and MPs.

At the interpersonal level, preparatory discussions about the collaborative relationship were held to establish clarity around roles and the scope of practice. Some practices formulated their collaboration in a written collaborative agreement, which NPs thought to be a *“source of clarity” (NP).* Medical practitioners with a good understanding of the role stated that the role had been well explained to them in advance, either by the NP or their medical association, which provided NP job descriptions. Following these preparatory measures, regular communication measures developed for the day-to-day running of the collaborative practice models.

Various communication methods were used to make up for the lack of direct interactions between NP and MP including an internal messaging system and informal face-to-face conversations, described as *‘talk in the corridors’* or a *‘chat over coffee’*. Nurse practitioners and MPs considered regular meetings as ideal, but in their absence, the spontaneous conversations were considered satisfactory. *“It feels informal because it is here in the tea room and in between. But it’s sufficient” (NP)*. Two of the five sites held planned team meetings on a weekly or fortnightly basis. To enable team meetings and manage the busyness of clinicians, one practice introduced a rule that no patients would be booked over lunchtime and all staff could meet during lunch. *“So if you have somewhere where people can sit down and have that meal together or morning tea together or somewhere to sit, that enhances collaboration” (NP).* Observations confirmed that communication and lunch breaks were significantly longer and more common where participants had the opportunity to sit down together.

Besides working around practical challenges, individual attitudes towards collaboration were found to have a significant impact on the success of collaboration. Nurse practitioners knew that they had to integrate themselves in a *“non-threatening way” (MP).* A nurse practitioner stated: *“You don’t try to take over. That would be a bad thing. And that would make us very unpopular”.* Accordingly NPs developed a strategy of careful negotiation within the MP’s domain of patient care. A NP described that she approached the MP in the practice whose idea of patient care was most consistent with hers in a particular case. Thus she found a way of getting approval for care without offending any of the MPs. *“I think, there is a little bit of … I don’t want to say manipulation… umm…a bit of selective choosing (laughs)” (NP).* It seemed NPs found a strategy of cautious confidence, which allowed them to make autonomous decisions and appear confident but not over-confident in their behaviour.

Nurse practitioners and MPs agreed that collaboration worked because of their trustful and respectful relationship. Developing trust through positive experiences contributed to diminished MP concerns. *“I’m just one of these older GPs who have gone from being totally opposed to the idea of nurse practitioner to being a complete convert” (MP).* Nurse practitioners reported that after some time MPs transferred tasks to the NP as a sign of increased trust. *“They [MPs] have expanded what they are happy for me to do” (NP).*

Commitment of individuals was important. Collaborative practice models in this sample worked because most MPs were willing to take a financial risk by working in collaboration with NPs for the advantage of better patient care. *“It is an important part of our practice, so I think, we should do it, even if it’s not a money making thing” (MP).* Considering the restrictions through Medicare policy and legislation, MPs as well as NPs were well aware that the collaboration models in the private sector existed because of the willingness of MPs. *“Collaboration between nurse practitioners and doctors depends on […] whether the owner of the practice is willing to do that or not” (MP).*

### Findings in comparison with existing models of collaboration

A majority of dimensions of the two theoretical models overlapped with the findings in this study (Table 2). Strong evidence of the importance of mutual trust and respect, compatible role perceptions, communicative behaviour and infrastructure for information exchange, shared goals and decision-making for collaboration were identified in both theoretical models [42, 43] and at sites in this study. Likewise formalisation tools such as policies, protocols and agreements, understood as structural factors affecting collaboration, were found in this study and in the earlier models.

Aspects of role enactment were mostly addressed in Corser’s model of nurse-physician interaction [42]. Personality, willingness and personal values as well as traditional role patterns and power symmetry were identified as having a strong influence on the functioning of collaboration in the current study. However, conditions of power symmetry were largely impeded by system structures and to a smaller extent by traditions of professionalisation and traditional gender or role norms as described by Corser [42].

Three dimensions developed by D’Amour et al. were only marginally present at the five sites in our study [43]. First, D’Amour et al. defined connectivity as a connection between individuals and the organisation based on feedback systems, meetings and committees to allow rapid coordination and adjustment of practice [43]. Practice adjustments and opportunities for meetings appeared to be easier to establish at smaller sites where meetings occurred frequently compared to larger sites. However, some participants at large sites and the community centre stated that support from the management level was important for the establishment of the collaborative practice model.

Second, centrality, described as authorities that provide clear directions [43] including professional boards, associations or government institutions, were only of marginal impact in our study. A nurse practitioner expressed her frustration with vague directions by authorities. *“I asked the nurses’ board about that [access to PBS] and they weren’t clear” (NP).* It is important to note that D’Amour’s Structuration Model was developed in Canada, where ‘health authorities’ govern the provision of healthcare in designated areas [43]. In Australia, a similar approach with local support for PHC institutions, Medicare Locals, were established in 2011 but a review in 2014 stated low functionality of these authorities [58]. In addition, centrality might play a larger role in inter-organisational collaboration, a focus of the Structuration Model but not of our study.

The third dimension, for which only limited evidence was found, is the influence on collaboration through the presence of a leader of collaboration. None of the participants identified a team member with such a position or role. However, as outlined in theme three, the practice manager played an important coordinating and organisational role in some of the collaborative practice models.

Our study identified two additional factors influencing collaboration not included in the two theoretical models. First, the consequences of NP autonomy on role enactment might be a particular problem for NPs and MPs but were not found to be a problem between other professions or organisations [43] or between general nurses and MPs [42], where lines of authority might be clearer. Corser [42] touched on the issue of autonomy with the dimension of power dynamics. Second, fiscal systems influenced the functioning of collaboration. Corser [42] as well as D’Amour and colleagues in their publications [43, 59] acknowledged that economic constraints and resources influence processes of collaboration but did not consider them as an extra dimension in their models.

## Discussion

This study investigated the experiences and perceptions of NPs and MPs in relation to collaborative practice in five PHC settings in Australia following amendments of policies regarding collaborative arrangements and NP access to healthcare services subsidy schemes. Although system structures were the main impediment to establish sustainable collaborative practice models, the willingness of practitioners and their individual relationships partially overcame the effect of system restrictions. Practitioners were able to establish, adjust and accept new routines, noticeable in their moving back and forth between new and traditional roles. While questionnaire results indicated that NPs and MPs experienced both high levels of collaboration and satisfaction with the collaborative relationship, and held strong beliefs in the benefits of collaboration the qualitative results revealed a more ambivalent picture of NPs’ and MPs’ experiences of collaboration. Financial issues as well as NP autonomy and have an impact on collaboration and expand existing theoretical models.

### Collaborative working within policy frameworks and existing infrastructure

Financial issues are a significant influence on collaboration in Australia by disadvantaging NPs in collaborative practice. Nurse practitioners receive lower rates of reimbursement than MPs for patient consultations, and only a limited number of Medicare items are available to them [60]. Differences in reimbursements rates for NPs and MPs reported from an economic case study of an Australian general practice corroborate our findings [61]. However, practitioners both in our study and in the USA highly valued NP access to a health insurance scheme as an enabler of collaborative practice models [27, 62, 63].

Study participants critiqued the fee-for-service model as negatively influencing collaborative practice. North American research supports our finding. A survey of 20,710 Canadian MPs showed that MPs working in a fee-for-service model were significantly less likely to collaborate with NPs [64]. An ethnographic study of three PHC teams in the USA identified fee-for-service models as a disincentive for health professionals to discuss mutual patient cases in the absence of a patient because it solely reimburses practitioners for face-to-face consultation time with patients [65]. The insecurity over financial benefits from collaborative practice inhibits supportive MPs from collaborating with a NP. Australian health care reformers missed a chance to learn from countries where NPs operate on a more sustainable level through targeted government initiatives to support team care approaches [27, 66]. For example, initiatives in Canada and the USA included incentive payments for MPs to join healthcare teams and government funded NP positions [67–69]. Such initiatives foster shared care of patients.

The Australian determination underpinning collaborative arrangements added to the power imbalance between NPs and MPs in collaborative practice models. Nurse practitioners in our sample valued the consultation availability of MPs but questioned the legal determination for two reasons. NPs considered it self-evident that they would consult another health professional if necessary and their choice of work location relied on the agreement of a collaborating MP. Consequently, NPs were in a dependent relationship [70] and disadvantaged in negotiating business terms such as income, leave regulations or payment for administrative support [71]. A literature review about collaborative arrangements in the USA concluded that mandatory collaborative arrangements hindered NP practice in areas of need or remote areas where no MPs are available or willing to enter a collaborative arrangement [72]. A cross-sectional analysis from 2001 to 2008 of 41 USA states showed that restrictive collaborative practice arrangements limited growth of NP numbers by 25 % [73]. Where system-level policies restrict NPs in their choice of practice and force them to practice below their potential, care resources are underutilised [74, 75].

Legal liability can be unclear in team structures [76]. Australian legislation underpinning collaborative arrangements appears to have added to the confusion about such liability [77, 78]. Study participants held diverse views about their accountability for patients who were jointly looked after by an NP and a MP reflecting lack of clarity about such liability. The current determination of Australian collaborative arrangements draws MPs into a commitment of “collaborative” working with a NP with poorly understood implications for practice. Medical practitioners may carry vicarious liability, where they are employers or in some cases practice owners, that is, they may be held accountable for the NP’s negligent action [79]. Thus MPs may be wary about entering collaborative arrangements and providing support for patients they have not seen.

Legal liability may be clearer without the legal requirement of collaborative arrangements [78]. Battaglia proposed complete practice independence for NPs so that “a practicing NP would generally bear the full liability for instances of malpractice arising from care provided by that NP” ([78], p. 1151). Resnick and Bronner emphasise the importance of outlining the scope of practice of NP and MP, communication and referral mechanisms in writing [80]. However, such agreements do not have to be linked to legislation and the current Australian determination fails to clarify legal liability.

Organisational structures contributed to the lack of equality between NPs and MPs in this study. The lack of space for NPs in PHC settings has been identified as a problem in a case study of three PHC sites in Canada [81] and in interviews with 16 NPs practicing in PHC settings in the USA [82]. It appeared MPs were given priority for offices and resources, which researchers described as “structural discounting” ([83], p. 90) of NPs.

Disruptions to existing routines, identified in this study in the form of interruptions to patient consultations and communication flow, were highlighted by Greenhalgh as a challenge for collaborative working [84]. Our findings support those from a Canadian ethnographic study of three multiprofessional PHC teams in which a lack of communal space and clinician time constraints impeded frequent meetings [85]. However, face-to-face meetings have been consistently reported as one of the most important features of collaboration because they guarantee exchange of ideas and information with immediate feedback when needed [65, 86, 87]. Consequently, the *“corridor conversations” (NP)* and a *“chat over a cup of coffee” (MP)* became significant routines for information exchange.

### Working collaboratively with co-existing roles

The addition of NPs to PHC sites required changes to existing role hierarchies, resulting in the co-existence and blurring of professional roles. A systematic review of studies across all types of healthcare settings reported that the combination of task delegation, substitution and complementarity in NP-MP teams added to the complexity of blurred role boundaries between NPs and MPs [76]. We found that NPs and MPs operationalised collaborative practice with overlapping and complementary roles. Roles overlapped when the NP adopted medical skills in her autonomous patient consultation and they complemented each other in joint patient consultations. The blurring of roles only emerged as a problem when legal and fiscal policies were difficult to apply in clinical practice.

Role theory can help to explain the traditional behaviour of some NPs and MPs. It is assumed that “persons are members of *social positions* and hold *expectations* for their own behaviors and those of other persons” ([88], p. 67). In our study, NPs and MPs worked in distinct nursing and medical roles because these were in line with their identity of nursing and medical care, based on “internalized role expectations” ([89], p. 286). Consequently, the identity of MPs can be linked to their socialisation as silo-workers [90]. Canadian researchers also found that MPs rarely consulted with NPs, even after an intervention addressing collaborative working of NP-MP teams in PHC [75]. We assume that one-sided consultation patterns from NPs to MPs in our study can be partially explained by the fact that MPs had not needed communication or collaboration with other health professionals in the past.

For NPs in our study, a strong influence on their role and identity adjustment was based in the way they used their autonomy. Feminist researchers developed the term ‘relational autonomy’, claiming that autonomy is hardly ever absolute but context bound and linked with given structures [91]. Nurse practitioners in our study possessed relational autonomy in the sense that they were entitled to work as autonomous health practitioners *within* a framework of professional structures, policy restrictions and their individual level of confidence to make autonomous decisions. An example of NPs practicing with relational autonomy relates to those NPs who adopted a level of assertiveness that did not undermine the MPs’ position. Assertiveness and confidence of NPs have been reported as facilitators of collaborative working in a mixed methods study of NPs and MPs in long-term care homes in Canada [47]. In our study, unassertive behaviour, including MP involvement where not strictly required, by otherwise very confident and highly competent NPs, was used as a purposeful strategy by all six NPs to enter existing MP-dominated structures.

### Successful collaboration relies on the commitment of individuals

Considering the barriers for collaborative practice from existing systems, organisational structures and neglect from government agendas, collaboration between NPs and MPs in our sample appeared to exist through individual relationships and personal experiences. This accords with other studies that identified relationships and the personality of practitioners as significant factors for successful collaboration [82, 87, 92].

Collaborative practice models in the Australian PHC context would not exist without the personal commitment of NPs and MPs. Their willingness and ability to work around system barriers was based on the value they ascribed to their relationship. This was reflected in largely positive perceptions of the collaborative relationship in interviews and the questionnaire. Furthermore, in contrast to the Canadian Structuration model of collaboration [43] Australian PHC collaboration models were a bottom-up approach, driven by individuals who received limited support and governance through government and healthcare system structures as identified in the deductive analysis.

### Strengths and limitations

The inclusion of five different sites spread across four Australian states generated a broad perspective on collaboration based on a multi-method dataset. The similarity with other research and theoretical models strengthened the credibility of findings and suggests their transferability within the Australian context of PHC, whilst noting that findings from case study research cannot be directly generalised to the general population of NPs and MPs.

Participating sites had well-established patterns of working together and recruitment of a negative or disconfirming case [36, 44] would have been a valuable addition to the sample. However, while we attempted to include sites with obvious inter-professional challenges, none were willing to participate. The recruitment of well-functioning teams was partly balanced out by participant statements about negative experiences in previous practices.

### Recommendations for practice, policy and research

While this study was conducted in the Australian setting, similarity with international experience suggests that recommendations coming from this study are relevant to health professionals in other countries where NP roles are being implemented.

The influence of existing policies on the success of collaborative practice models needs consideration. Reimbursement structures for NPs have to ensure financial viability of NPs in PHC to increase the motivation for MPs to work in collaboration. For example, NPs should be granted access to a similar range of MBS items currently available for MPs, including procedure-based items (e.g. conducting and interpreting electrocardiography and spirometry, ordering female pelvic ultrasounds and suturing wounds) in addition to time-based consultation items. Further funding for collaborative practice models may come from private health funds if they reimburse patients who use NP care services. In line with trends in the USA, mandatory collaborative arrangements for NPs should be removed from legislation to facilitate autonomous NP practice and to minimise blurring of legal liability.

Improvements in infrastructure and practice level arrangements are recommended to facilitate NP-MP interaction within practice settings. Opportunities for face-to-face meetings should be enhanced because face-to-face conversations were the most valued mode for information exchange. Regular meetings can serve as an occasion to address practical issues between participants, to foster information exchange about mutual patients and increase mutual learning. Where scheduled meetings are not possible, opportunities for informal conversations can be enhanced through communal areas and facilities where this is possible. Practice managers should be utilised for their potential leadership role in fostering collaboration. Nurse practitioners should be given access to space and resources that equal the MP’s access to infrastructure, including office space. Preparatory clarification of scope of practice, consultation and referral mechanisms as well as roles and responsibilities is recommended. It appeared useful for practitioners to put this agreement in writing (on a voluntary basis and not based on legislative requirements) and to address liability of practitioners for different scenarios such as 1) patients seen together; 2) patients seen by only one practitioner but advice was given by another practitioner (by phone, email, face-to-face conversation); and 3) NPs working under vicarious liability, when the employer (MP) may hold some responsibility for the employee (NP).

Most patient consultations occurred in separate sessions affirming that NPs are autonomous healthcare providers. Future research could investigate frameworks within which NPs are able to establish their own businesses. This study showed that the dependence on MPs and low reimbursement rates made it difficult for NPs to establish their own clinics in Australia.

## Conclusions

These findings represent the experiences and perceptions of NPs and MPs in collaborative practice models following the introduction of new policies in the Australian setting regarding NP access to the public health insurance scheme and collaborative arrangements. Numerous challenges posed by system structures at policy and practice level and differing perceptions of role enactment were identified. Findings provided an understanding about the difficulty of NPs to enter existing healthcare systems and help to understand some reservations of MPs towards collaboration with NPs. Nevertheless with their willingness and ability to modify routines and roles and accept existing structural frameworks, NPs and MPs were able to establish well-functioning models of collaboration. The individual determination of practitioners to make it work was crucial for the implementation of these models of care because their establishment was challenging at those sites where external support by government agencies was lacking. The evidence-base from this study on collaborative practice models in Australian PHC settings will facilitate new discussions with policy makers, healthcare funds, medical and nursing associations, politicians and key stakeholders who influence healthcare reform.

## Abbreviations

ACNP, Australian College of Nurse Practitioners; AHPRA, Australian Health Practitioner Regulation Agency; FFS, fee-for-service; GP, general practitioner; MBS, medicare benefit schedule; MMR, mixed methods research; MP, medical practitioner; NP, nurse practitioner; PBS, pharmaceutical benefit scheme; PHC, primary healthcare; PM, practice manager; UK, United Kingdom; USA, United States of America; WHO, World Health Organisation

## Additional files

Additional file 1: Interview Schedule for Nurse Practitioners (PDF 71 kb) Additional file 2: Interview Schedule for Medical Practitioners (PDF 72 kb) Additional file 3: Interview Schedule for Practice Managers (PDF 341 kb)
