# Supplementary material for: Experiences of nurse practitioners and medical practitioners working in collaborative practice models in primary healthcare in Australia – a multiple case study using mixed methods
Source: BMC Fam Pract. 2016 Jul 29;17:99. doi: 10.1186/s12875-016-0503-2 (PMC4966821; doi:10.1186/s12875-016-0503-2)
Supplement: Additional file 1: — Interview Schedule for Nurse Practitioners (PDF 71 kb) [file 12875_2016_503_MOESM1_ESM.pdf]

Interview Schedule for Nurse Practitioners

1. Introductory questions around role, type of patients, employment status.

2. Who initiated the process of introducing a NPs to this practice?

3. How would you define collaboration?
   (- What are essential elements of collaboration between NPs and MPs?)

4. How would you describe someone who works collaboratively?
   (collaborative behaviour)

5. Please describe to me some situations where you collaborate with the MP?
- Meetings, consultations, referrals
  (How do you communicate in the patient's notes?)

6. From your experience, what works well in this practice in regards to collaboration between NPs and MPs?
   (- What do you consider facilitators for collaboration in this practice? Can you give me some examples?)

7. What does not work so well?
   What could be improved?
   (Please describe to me some of the challenges for you working in collaboration in this practice?)
   (What do you think are the barriers to collaborative working with MPs?)

8. Are there practice features in place that streamline/foster collaborative care? If so what are they?

9. How is the decision made about who of you will see a patient?

10. How do you decide together on a patient's treatment?
- Who is liable for the patient care?

11. What would you advise others to do to enhance collaborative working with MPs, if they are doing this for the first time?

12. Please tell me what you know about the collaborative arrangements as required by the Government for NPs to access MBS and PBS items.
    - What are your thoughts about the collaborative arrangements in this practice? Are they helpful or not helpful for your practice as a NP and why?

13. How does NP prescribing take place in this practice?

14. How is your position funded?

15. How does autonomous practice for the NP work in this practice?

16. Did you have interdisciplinary units (shared classes with other health professionals?) - Where have you been trained?
