# Supplementary material for: Experiences of nurse practitioners and medical practitioners working in collaborative practice models in primary healthcare in Australia – a multiple case study using mixed methods
Source: BMC Fam Pract. 2016 Jul 29;17:99. doi: 10.1186/s12875-016-0503-2 (PMC4966821; doi:10.1186/s12875-016-0503-2)
Supplement: Additional file 2: — Interview Schedule for Medical Practitioners (PDF 72 kb) [file 12875_2016_503_MOESM2_ESM.pdf]

Interview Schedule for Medical Practitioners

1. Introductory questions around role, type of patients, employment status.

2. Who initiated the process of introducing a NPs to this practice?

3. How would you define collaboration?
   (- What are essential elements of collaboration between NPs and MPs?)

4. How would you describe someone who works collaboratively?
   (collaborative behaviour)

5. Please describe to me some situations where you collaborate with the NP?
- Meetings, consultations, referrals
  (How do you communicate in the patient's notes?)

6. From your experience, what works well in this practice in regards to collaboration between NPs and MPs?
   (- What do you consider facilitators for collaboration in this practice? Can you give me some examples?)

7. What does not work so well?
   What could be improved?
   (Please describe to me some of the challenges for you working in collaboration in this practice?)
   (What do you think are the barriers to collaborative working with NPs?)

8. Are there practice features in place that streamline/foster collaborative care? If so what are they?

9. Did you experience any changes in the practice or for yourself through the collaboration with the NP?

10. How is the decision made about who of you will see a patient?

11. How do you decide together on a patient's treatment?
    Who is liable for the patient care?

12. What is your opinion on autonomous NP practice? (Advantages/disadvantages?)

13. Is it different collaborating with a NP compared to collaborating with other health care professionals? In what way?

14. What would you advise others to do to enhance collaborative working with NPs, if they are doing this for the first time?

15. Have you heard about the collaborative arrangements, required by the Government for NPs to access MBS and PBS items.
    - What are your thoughts about the collaborative arrangements in this practice?

16. Are you involved in prescribing practices undertaken by the NP? If so, how?

17. Did you have interdisciplinary units (shared classes with other health professionals?)
    - Where have you been trained?
