# Supplementary material for: Experiences of nurse practitioners and medical practitioners working in collaborative practice models in primary healthcare in Australia – a multiple case study using mixed methods
Source: BMC Fam Pract. 2016 Jul 29;17:99. doi: 10.1186/s12875-016-0503-2 (PMC4966821; doi:10.1186/s12875-016-0503-2)
Supplement: Additional file 3: — Interview Schedule for Practice Managers (PDF 341 kb) [file 12875_2016_503_MOESM3_ESM.pdf]

Interview Schedule for Practice Managers

1. Introductory questions around practice setting, staff and role, employment status

2. Who initiated the process of introducing a NPs to this practice?

3. How would you define collaboration?
   (- What do you understand about the meaning of collaboration?)

4. How would you describe someone who works collaboratively?
   (collaborative behaviour)

5. Tell me about your experience of working in a collaborative practice. What works well and what does not work so well?

6. Please describe to me some situations where the MP and the NP work collaboratively?

- Meetings, consultations, referrals

7. Does your role as practice manager facilitate NP-MP collaboration? If so, how?

8. Are there practice features in place that streamline/foster collaborative care? If so what are they?

9. Please describe to me some of the challenges for the NPs and MP working in collaboration in this practice?

10. What do you consider facilitators for collaboration in this practice? Can you give me some examples?

11. How does autonomous practice for the NP work in this practice?
    - Examples?
